# Investigating the Use of Mobile Health Interventions in Vulnerable Populations for Cardiovascular Disease Management: Scoping Review

**DOI:** 10.2196/14275

**Published:** 2019-10-07

**Authors:** Sahr Wali, Neesha Hussain-Shamsy, Heather Ross, Joseph Cafazzo

**Affiliations:** 1 Institute of Health Policy, Management and Evaluation Dalla Lana School of Public Health University of Toronto Toronto, ON Canada; 2 Centre for Global eHealth Innovation Techna Institute University Health Network Toronto, ON Canada; 3 Ted Rogers Centre for Heart Research University Health Network Toronto, ON Canada; 4 Department of Medicine University of Toronto Toronto, ON Canada; 5 Peter Munk Cardiac Centre University Health Network Toronto, ON Canada; 6 Institute of Biomaterials and Biomedical Engineering University of Toronto Toronto, ON Canada

**Keywords:** mobile health, health services, indigenous, low- and middle-income countries, cardiovascular disease, self-care

## Abstract

**Background:**

Cardiovascular disease (CVD) has grown to become one of the leading causes of mortality worldwide. The advancements of CVD-related treatments have led to a decline in CVD prevalence among individuals in high-income countries (HICs). However, these improvements do not reflect the state of individuals in low- and middle-income countries (LMICs) and vulnerable subgroup populations in HICs, such as the Indigenous. To help minimize the health disparities in these populations, technology-based interventions have been offered as a potential solution, but there is concern regarding if they will be effective, or even needed, as these tools have been designed for use in HICs.

**Objective:**

The objective of this study was to explore how mobile health (mHealth) interventions currently assist individuals in Indigenous communities and LMICs with CVD management.

**Methods:**

A scoping review guided by the methods outlined by Arksey and O’Malley was conducted. A comprehensive search was completed by 2 reviewers in 5 electronic databases using keywords related to mobile health, cardiovascular disease, self-care, Indigenous communities, and LMICs. Studies were screened over 2 rounds and critically reviewed using a descriptive-analytical narrative method. Descriptive data were categorized into thematic groups reflecting the major findings related to the study objective.

**Results:**

We identified a total of 11 original articles and 11 review papers that met the criteria for this scoping review. The majority of the studies included a telemonitoring- and text messaging (short message service, SMS)–related feature associated with the intervention. The use of SMS was the most common approach to effectively promote disease management among individuals in both LMICs and Indigenous communities. However, customizing for cultural considerations within the design of the intervention was highlighted as a pivotal component to encourage CVD management. Specifically, individuals emphasized that the inclusion of collaborative partnerships with community members would strengthen the effectiveness of the intervention by ensuring it was designed with the appropriate context.

**Conclusions:**

Technology-based interventions used within Indigenous communities and LMICs have shown their potential to assist individuals with managing their condition. Although the literature available regarding this topic is limited, this review outlines key components to promote the effective use of these tools in the context of these vulnerable populations.

## Introduction

The World Health Organization has indicated that cardiovascular disease (CVD) is the leading cause of mortality, accounting for nearly 31% of all deaths worldwide [1-3]. Despite the increase in prevalence of the disease, CVD is largely treatable or controllable if patients are able to properly manage and monitor their symptoms [2,4]. Previous studies have found that the promotion of CVD self-care led to improved cardiovascular outcomes [2-4]. In Canada, the dissemination of CVD self-care strategies and clinical practice guidelines led the rate of CVD to decline among most age groups [4,5]. However, these improvements did not reflect the state of more vulnerable and at-risk populations within the country, such as the Indigenous [4,5].

Indigenous populations across various high-income countries (HICs), such as Canada, the United States, and Australia, have reported that they have experienced a rise in CVD prevalence and associated mortality, relative to the rest of the population [3-6]. The discrepancy in CVD improvement across HICs is largely linked to poor disease control and disparities in social determinants of health [2,4,7]. This includes factors such as housing, education, access to health services, and income [4].

Many of the risk factors affecting Indigenous populations are shared by individuals from low- and middle-income countries (LMICs) [4,8]. The effect of these conditions is supplemented by the lack of preventative strategies and health care resources available for both populations [2,3]. Indigenous populations do not receive or have access to the same level of care for the treatment or management of CVD as non-Indigenous populations [9]. Consequently, they are 3 times more likely to die from a CVD-related event [4,9]. Similarly, there are currently 17.5 million CVD-related deaths occurring annually, and more than 75% of the CVD-related deaths are accounted by LMICs because of the lack of clinics and assistive tools available for appropriate diagnosis and care [8]. Both these populations may have differences in their history, culture, and structure of their health care system, but the underlying shared causes leading to poor patient outcomes (ie, geographic and socioeconomic factors) create a comparable link between the 2 populations. Thus, as both Indigenous communities and individuals with CVD living in LMICs face challenges involving accessibility to appropriate resources, more innovative interventions promoting CVD management within their environmental setting need to be introduced [4,10].

With the widespread penetration of mobile phones, mobile health (mHealth) technology offers a promising platform to support CVD management in populations with less health resources [8,11]. It is estimated that more than 60% of individuals in LMICs own a mobile phone and up to 85% have access to a device [12]. mHealth apps could serve as a low-cost tool to simplify and promote CVD management [13-15]. Previous studies have shown that mHealth tools and other technology-related interventions have had a positive impact on improving patient outcomes [13-16]. This has been supplemented by its ability to simplify or automate self-care steps as well as provide additional support and guidance from a health care professional [14-16]. However, as the majority of mHealth interventions have been designed for use in HICs, there is concern regarding whether these tools will be effective in low health resource settings [15]. To address this gap in knowledge, this review aims to study how various mHealth interventions (ie, apps, text message, and telemonitoring) assist Indigenous communities and individuals in LMICs with CVD management (ie, self-care and remote management).

## Methods

### Review Framework

This review was guided by Arksey and O’Malley’s 5-stage scoping review framework: (1) identifying the research question, (2) identifying relevant studies, (3) selection of studies, (4) charting the data, and (5) summarizing and reporting the results [17]. Their review approach synthesizes and maps key concepts from the literature available to give a better understanding of the impact of innovative self-care tools in more vulnerable populations.

#### Research Question

The focus of this review was to explore how mHealth interventions for CVD management assisted individuals from Indigenous populations and LMICs. mHealth tools are often evaluated in HICs; thus, this led to the following guiding question: *What is known in the literature about the use of mHealth interventions on CVD management in Indigenous communities and LMICs?*

#### Search Strategy

A preliminary scan of literature was conducted on 2 academic databases (MEDLINE and EMBASE) with the following search terms: mobile health, cardiovascular disease, self-care, Indigenous, and LMIC. Keywords and related subject headings were refined according to text contained in the title and abstract of the initial literature search (Textbox 1). On the basis of the keywords identified, 2 reviewers (SW and NHS) independently conducted a comprehensive search in 5 electronic databases: MEDLINE, EMBASE, Web of Science, Cumulative Index to Nursing and Allied Health Literature, and Scopus. Reference lists were also reviewed to extract additional studies that were not found in the initial search. This review did not restrict studies according to the year of publication, but studies were considered ineligible if they were not published in English.

**Scoping review search strategy.**

**Scoping review keywords:**

1. Mobile health

- Mobile health OR mHealth OR digital health OR health application* OR health app* OR health technolog* OR eHealth OR mobile phone* OR phone-based OR SMS OR short message service* OR text message* OR telehealth OR telephone monitor* OR telemedicine

*AND*

2. Cardiovascular disease

- Cardiovascular disease* OR CVD OR heart failure OR HF OR stroke* OR heart attack* OR chronic disease* OR hypertension OR HT

*AND*

3. Self-care

- Self-car* OR disease manag* OR self manag* OR remote manag* OR remote car* OR manag* OR treatment

*AND*

4a. Indigenous

- Indigenous OR Aboriginal* or Metis OR Inuit* OR First Nation* or Native American*

*OR*

4b. LMIC

- LMIC OR low income OR middle income OR developing countr* OR third world countr* OR vulnerable population*)

#### Study Selection

The retrieved literature was screened over 2 rounds for study selection. In the first round, titles and abstracts were reviewed according to the inclusion and exclusion criteria listed in Textboxes 2 and 3, respectively. In the second round, abstracts and the full text were screened to determine if they met the outlined criteria.

**Inclusion criteria for study selection.**

*Inclusion criteria:*

- Primary intervention involves mobile health– or technology-related tool or aid for self-care
- Distinction of an Indigenous or low- and middle-income country population—study could include nonvulnerable population in addition to Indigenous population or individuals in low- and middle-income country
- Study population has at least one cardiovascular disease–related condition (ie, heart failure, stroke, or heart attack)

**Exclusion criteria for study selection.**

*Exclusion criteria:*

- Intervention does not include cardiovascular disease management, monitoring, or promotion as a key component in the study
- Primary study population is high-income country
- Grey literature, review papers, or study protocols

#### Charting and Extracting Data

Articles meeting the inclusion criteria were critically reviewed using Arksey and O’Malley’s descriptive-analytical narrative method [16,17]. Data extracted included the year of publication, study location, intervention type (phone, tablet, telemedicine, etc), study population, aim of study, methodology, outcome measures, and main findings.

#### Summarizing and Reporting Results

A numerical analysis of the extent and nature of the studies was conducted using tables and chart mappings. The descriptive data were analyzed using conventional content analysis. In accordance with the user-centered design framework, 2 reviewers (SW and NHS) examined the descriptive data and identified codes relative to the findings [16]. These codes were then organized into thematic groups to summarize the literature according to their main findings and present a narrative relating to the research question.

## Results

### Common Themes

**Figure 1:**
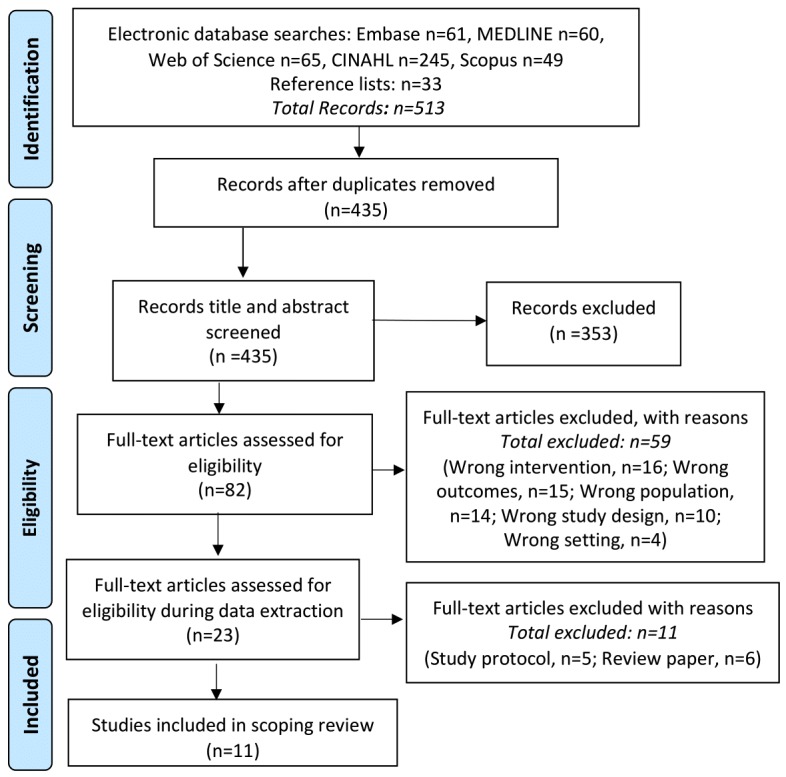
Systematic scoping review search strategy—Preferred Reporting Items for Systematic Reviews and Meta-Analyses flowchart. CINAHL: Cumulative Index to Nursing and Allied Health Literature.

A total of 513 articles were identified from the 5 databases and reference lists searched. Furthermore, 78 duplicate articles were removed, and the remaining 435 articles were screened according to the inclusion and exclusion criteria listed in Textboxes 2 and 3, respectively. After a review of the title and abstracts, 82 articles were included for the full-text review. Moreover, 59 articles were excluded during the full-text screening because of a series of implications with the inclusion and exclusion criteria outlined in Figure 1. A total of 11 articles were included in the scoping review (Figure 1). However, owing to the limited amount of studies found, the review papers and study protocols meeting the inclusion criteria were reviewed separately for common themes.

#### Findings From Original Studies

From the 11 articles, the most common study locations were Latin America (number of articles [n]=3), New Zealand (n=2), and South Africa (n=2). However, the majority of the study populations were LMICs (n=8; Multimedia Appendix 1). The interventions used within various studies were differentiated by the combination of their use of (1) short message service (SMS) text messaging and telemonitoring (phone calls; n=3), (2) telemonitoring only (n=4), and (3) SMS text messages only [3]. An article did not have an intervention outlined as it was a qualitative study focused on identifying the barriers and opportunities for technology in Indigenous populations [18].

The aim of 10 of these studies was to evaluate the outlined interventions’ ability to improve disease management. This included the impact of a reminder system (n=6), health education (n=2), and telemonitoring (n=6). A total of 8 of the studies commonly used a behavior change or the satisfaction of using the intervention as their primary outcome measure (Multimedia Appendix 1).

The main findings from these studies indicated that the various mHealth interventions promoted a positive behavior change for CVD management. This included improved medication adherence (n=4), monitoring and control of systolic blood pressure (BP; n=4), and overall self-care (n=5). Of the articles, 6 included an SMS component to the intervention, and in 5 of those studies, patients explicitly stated that they preferred the SMS text messages as a reminder system. In 2 studies, patients using the self-care program reported improvements in their satisfaction with their care and overall quality of life compared with the control group.

#### Findings From Review Papers or Study Protocols

Of the selected articles, 6 were review papers (4 systematic reviews and 1 literature review and 1 rapid review), 4 were randomized controlled trial (RCT) protocols, and 1 was a co-design paper (Multimedia Appendix 2, [19-21]). The majority of these articles’ study locations were in Australia (n=5), and of the 11 articles, there was an even distribution between the study populations (5 LMIC, 5 Indigenous, and 1 LMIC and Indigenous).

The interventions included within the articles varied by the following components: (1) mHealth app and SMS text message (n=4), (2) mHealth app and educational program (n=1), (3) mHealth app and telemonitoring (n=3), and (4) mHealth app only (n=3). The aim of these articles was to provide evidence on the effect mobile phone–based solutions had on CVD management within either population (n=6). Out of 4 of the review articles, 3 found that mHealth tools were a sufficient method to promote CVD management, specifically with the use of SMS text messages. In 3 articles, community collaboration and cultural sensitivity were also highlighted as key components for intervention success.

#### Findings From Thematic Analysis

In total, 2 dominant themes related to the design of mHealth intervention and its resultant effect were observed: (1) establishing a reminder system and (2) customizing for cultural considerations.

##### Establishing a Reminder System

Medication management was found to be the most common form of CVD-related self-care in LMICs and Indigenous communities [22-32]. In 2016, Gu et al [18] found that the issues contributing to poor medication adherence in the Australian Indigenous communities included poor patient knowledge of medication effects, cost-value of medication use, and general forgetfulness. Multiple studies indicated that mHealth solutions promoted medication adherence by acting as a reminder system [22-32].

Specifically, the combination of SMS text messages and telemonitoring was shown to be effective in improving BP control and overall treatment adherence [22,24]. In a study by Sarfo et al [33], they introduced a phone-based intervention that included Bluetooth telemonitoring of BP, SMS text messages, and nurse guidance for HF self-care. After 9 months of the intervention’s implementation, patients in the intervention group reported higher medication adherence scores and better systolic BP control (73.3% of patients with systolic B*P*<140 mmHg) compared with patients in the control group (43.3% of patients with systolic B*P*<140 mmHg) [33].

Telemonitoring alone also had a significant effect on improving disease management [26-28]. In 2012, Piette et al conducted an RCT to test the effectiveness of a self-management system that included telemonitoring, behavior change calls, and home BP monitoring [28]. They found that patients receiving the intervention had a reduction of 4.2 mmHg in their BP compared with the control group [28]. Patients had also reported that the intervention increased their medication adherence and satisfaction with their care. In 2016, Piette et al tested the same platform in Bolivia with the addition of a CarePartner feature, where a family or friend would receive a summary of the patient’s status and guidance on how to support their self-management [26]. Patients receiving the intervention had significant improvements in their self-care reports, especially among Indigenous and low-literacy patients [26].

The use of SMS text messages alone was noted as the most common and effective method to increase medication adherence in LMICs compared with smartphone apps [34,35]. In a study by Kamal et al [24], they evaluated the effect of SMS medication reminders on recent stroke survivors in Pakistan. Results indicated that patients receiving the intervention had a 4.09 times lower risk of being low adherent [24]. In a study by Hacking et al [23], they found similar results, as SMS text messages improved self-reported CVD management. In addition, their study found that patients preferred the SMS text messages as a reminder system more than education.

##### Customizing for Cultural Considerations

Among the target populations, the inclusion of unique cultural considerations was identified as a key component for the promotion of CVD management [35,36]. In 2016, Gu et al [18] conducted a participatory action focus group, and they identified that technology-based solutions in Indigenous communities would need to be both culturally and literacy sensitive. Banbury et al [37] explained that Indigenous populations already had a *preset fear of nontraditional tools*; therefore, new technologies would need to be developed in partnership with the community to ensure they reflected the populations’ needs.

Bradford et al [9] collaborated with the Mayor and Council of a remote Aboriginal Community to customize their existing mobile phone cardiac rehabilitation health program for Indigenous Australians. Following this study, the rehabilitation program was modified by simplifying the text and user interface, adding Indigenous artwork, and framing educational material in a culturally appropriate context.

## Discussion

### Principal Findings

The purpose of this scoping review was to identify how mHealth interventions were utilized in Indigenous populations and individuals in LMICs for CVD management. The amount of available literature exploring this topic was very limited as the target population for most studies was based in an HIC. However, based on the articles found, the use of mHealth solutions showed the potential to improve CVD management through the promotion of medication adherence and health education.

Throughout this review, more than half of the patients in the studies identified were classified as nonadherent [26]. The majority of Indigenous people and LMIC populations reside in remote areas that are limited in the health resources available [4,37]. The addition of telemonitoring and/or SMS text messaging allows patients unable to attend obtain medical assistance to actively manage their treatment at home [26-28].

The use of SMS text messages was more common than telemonitoring or other interactive methods within the target populations [22,23]. Regardless of its prevalence, phone calls were identified as a better alternative to promote disease management because of its ability to cater to patients with low health literacy and serve as more than just a reminder system by providing additional education [25-28]. With this, there is a concern that the use of SMS may not be as effective in promoting self-care as anticipated. However, in both SMS- and phone call–based interventions, the studies may have reported that medication adherence and patient self-care improved, but their effect size was only quantified in 1 study [24]. To determine the impact of each intervention on CVD management and other key outcome measures, further research is required.

The beneficial role of a caregiver, in addition to the use of an mHealth intervention, was also highlighted in this review. In a series of 3 studies conducted by Piette et al, they had tested the use of their self-management system (ie, telemonitoring, phone calls, and home BP management) and found that the addition of a CarePartner significantly improved patient self-care compared with the use of the mHealth intervention alone or standard care [26-28]. These findings support the idea that by collaborating with carers and partners within a self-care program, patient disease management can be improved. The literature supporting this topic for these specific populations is limited, but it provides an additional avenue for further study.

A key strength the studies highlighted involved their identification of cultural factors that needed to be considered within their design. Specifically, individuals in both Indigenous populations and LMICs commonly have low literacy levels. Their lack of knowledge or understanding of their medication results in compromising their ability to follow their treatment regimen. A few of the studies found were in the process of identifying factors that would need to be accommodated for within their intervention, and 1 study had already begun modifying their mHealth platform [9,18,36]. The importance of cultural factors was found to be more evident in Indigenous populations compared with LMICs. To further understand if there is a comparable link between both populations and their use of mHealth interventions for self-care, future research should look to explore what key determinants are responsible for promoting self-care.

#### Limitations

This scoping review was limited as most of the articles’ results were composed of patients’ self-reported data that might have been inaccurate. We also included review papers and protocol papers within this review to expand the breadth of studies analyzed, but this might have impacted the validity of our overall findings. In addition, only 3 out of the 11 original articles included an Indigenous study population. Moreover, 2 of those studies including the Indigenous population were not able to evaluate the impact of the intervention on patient outcomes, as one was a qualitative participatory action focus group and the other was focused solely on intervention feasibility.

#### Conclusions

The inequities present among Indigenous communities and LMICs contribute to the growing prevalence of CVD within their populations. mHealth interventions for disease management have primarily been implemented in HICs; however, simpler versions of these tools have been shown to improve CVD self-care in these more vulnerable populations. Cultural compatibility is essential for the success of these interventions in low health resource settings. Nevertheless, although the literature supporting mHealth in these populations is limited, their results indicate that they have a promising future.

## Appendix

Multimedia Appendix 1 Charting of scoping review—original studies.

Multimedia Appendix 2 Charting of scoping review—review and design methodology papers.

## Abbreviations

BP: blood pressure
CVD: cardiovascular disease
HIC: high-income country
LMIC: low- and middle-income country
mHealth: mobile health
RCT: randomized controlled trial
SMS: short message service
