# Supplementary material for: Investigating the Use of Mobile Health Interventions in Vulnerable Populations for Cardiovascular Disease Management: Scoping Review
Source: JMIR Mhealth Uhealth. 2019 Oct 7;7(10):e14275. doi: 10.2196/14275 (PMC6803887; doi:10.2196/14275)
Supplement: Multimedia Appendix 1 [file mhealth_v7i10e14275_app1.pdf]

Multimedia Appendix 1. Charting of scoping review—original studies.

Reference and location	Intervention type	Population	Aim of study	Outcome measures	Main findings
Bobrow et al, 2016 [19], South Africa	Mobile phone SMS[a] information for treatment adherence; mobile phone SMS information for treatment adherence and interactive *Please-Call-Me* requests	LMIC[b]	To assess the effect of automated SMS text messages on controlling BP[c]	Systolic BP	Mean systolic BP change was −2.2 mmHg for mobile phone SMS information for treatment adherence intervention and −1.6 mmHg for mobile phone SMS information for treatment adherence and interactive *Please-Call-Me* requests intervention. There was no evidence that the interactive intervention had a better effect
Gu et al, 2016 [18], New Zealand	Participatory action focus group	Indigenous (New Zealand Maori people)	To understand patient and provider perspectives on how eHealth[d] tools can support Indigenous population with high CVD[e] risk	Barriers for long-term medication adherence; technology opportunities for medication adherence promotion	Medication adherence issues related with patients' knowledge of side effects, doctor communication, costs versus value of medication, and general forgetfulness. Technology solutions should improve health knowledge (literacy sensitive) and patient-provider communication (training on cultural sensitivity) and reduce costs (telehealth and simplify prescription refill process) and forgetfulness (reminder system)
Hacking et al, 2016 [20], South Africa	SMS health education on hypertension and healthy lifestyle guidelines	LMIC	To determine if SMS health information would improve patient health knowledge and patient self-care	Health knowledge; self-reported behavior change	No change in health knowledge found. Self-reported behavior had positive increase in intervention group. Patients preferred SMS rather than the standard of care with physicians providing verbal information, as the SMS acted as a reminder system more than education
Kamal et al, 2015 [21], Pakistan	SMS reminders for stroke survivors in Pakistan	LMIC	To test the effectiveness of SMS reminders on improving medication adherence	Medication adherence—Morisky Medication Adherence Questionnaire; stroke risk factors—BP; intervention satisfaction	Intervention group had improved medication adherence scores compared with control group. Control group also had 4.09 higher risk of being low adherent. Mean diastolic BP was 2.6 mmHg lower in intervention group
Hemmati Maslakpak and Safaie, 2016 [26], Azerbaijan	3 groups: SMS reminder messages and educational content;	LMIC	To compare the effectiveness of SMS intervention	Medication adherence—Hill-Bone Medication	Medication adherence was found to be statistically significant for intervention groups compared with control group. However, medication adherence scores

	reminder cards and educational content; control-only educational content		with that of reminder cards to promote medication adherence	Adherence Scale	were not statistically different for SMS and reminder card groups (*P*>.05). Both SMS and reminder cards were suitable methods to promote medication adherence
Piette et al, 2016 [23], Latin America	Weekly monitoring and self-management support/education calls with tailored feedback based on the patient's responses; CP[f]-family/friend to assist patient; CPs receive patient status summary and guidance on how to support patient self-management	LMIC (Bolivia)	To determine if automated telephone feedback to informal caregivers increases mHealth[g] engagement and patient outcomes	Baseline medication adherence—Morisky Medication Adherence Scale; patient perceived health status; call completion	Patients in the intervention group were significantly more likely to report excellent health and less likely to report days in bed because of illness. The intervention group also had a higher call completion rate. Within the subgroups, the intervention group had tripled call completion rates for both Indigenous and low-literacy patients
Piette et al, 2014 [24], Latin America	Survey on mobile phone, health, and access to care; mHealth platform with weekly interactive automated telephone monitoring and self-management support	Indigenous and LMIC (Bolivia)	To determine the current state of patient phone use and access to care and evaluate how intervention affects chronic disease management	Mobile phone use; current language; call completion; self-care reports	37% patients spoke Indigenous languages; 82% had mobile phone and 45% used text messaging. Mobile phones were least common in Indigenous language–speaking patients. Call completion was lower in older adults, but no difference was found according to health care access or ethnicity. Excellent/good self-care reports increased as intervention program progressed. Patient-reported improvements were associated with better medication adherence
Piette et al, 2012 [25], Latin America	Automated self-management calls and home BP monitoring	LMIC (Honduras and Mexico)	To evaluate effect of behavior change calls and BP monitoring on hypertension management and CVD-related outcomes	Systolic BP; medication-related problems; satisfaction with care	Patients' BP in intervention group decreased 4.2 mmHg compared with control. The study reported that fewer medication problems and greater satisfaction with care was found in the intervention group. Patients with low literacy or significant needs had clinically significant improvement in BP
Riley et al, 2015 [27],	Mobile, broadband-enabled remote	Indigenous (Native	To assess feasibility of intervention	Health care utilization:	45 patients were enrolled in the study, and 57 patients declined to participate. HF patients enrolled

United States	monitoring device for HF[h] (app+Bluetooth scale+BP monitor+wireless finger pulse oximeter)	American Reserve)	to facilitate patient and care team comanagement of HF in rural and Native American patient population	hospital visits; days at hospital	in the study showed statistically significant reductions in health care utilization. These reductions were significantly greater compared with patients that declined to participate but not significant compared with patients in matched cohort
Sarfo et al, 2018 [30], Ghana	Phone-based intervention (SMS and Bluetooth telemonitoring of BP) under nurse guidance for self-care	LMIC (stroke patients)	To test the feasibility and efficacy of mHealth technology–enabled, nurse-guided intervention to improve BP control	Systolic BP; medication adherence—Morisky Medication Adherence Scale; self-regulation—Treatment Self-Regulation Questionnaire	67.7% of patients had systolic BP <140 mmHg at month 3 compared with 46.7% in the control group. Intervention group had greater medication adherence. This intervention was feasible and improved patient outcomes
Venter et al, 2012 [28], New Zealand	Home-based telehealth terminal: touch-screen computer to measure peripherals; machine to measure body weight, oxygen level, lung function, and BP; Web portal reviewed by local nurses and supported by clinical algorithm	Indigenous (Maori people)	To investigate acceptability and usefulness of intervention for chronic disease management for Maori people and others living in rural areas	Quality of life–36-item short form health survey; St George Respiratory Questionnaire and the K10 questionnaire; service utilization; usefulness interviews	Intervention group showed a consistent improvement in quality of life compared with control. Reduced hospitalizations were noted in both the control (19%) and telehealth (25%) groups. Participants indicated that the technology was acceptable to most patients and their families

[a]SMS: short message service.
[b]LMIC: low- and middle-income country.
[c]BP: blood pressure.
[d]eHealth: electronic health.
[e]CVD: cardiovascular disease.
[f]CP: CarePartner.
[g]mHealth: mobile health.
[h]HF: heart failure.
