# Supplementary material for: Investigating the Use of Mobile Health Interventions in Vulnerable Populations for Cardiovascular Disease Management: Scoping Review
Source: JMIR Mhealth Uhealth. 2019 Oct 7;7(10):e14275. doi: 10.2196/14275 (PMC6803887; doi:10.2196/14275)
Supplement: Multimedia Appendix 2 [file mhealth_v7i10e14275_app2.pdf]

Multimedia Appendix 2. Charting of scoping review—review and design methodology papers.

Reference and location	Intervention type	Population	Aim of study	Methods	Main findings
Abrahams-Gesse et al, 2018 [37], Argentina	Mobile phone CVD[a] risk calculator; electronic appointment scheduling system on app; educational and appointment reminder text messages	LMIC[b]—concentration on uninsured population	To determine if the mHealth[c] tool will promote attendance to appointments and increase follow-up visits for high-risk patients	Pragmatic RCT[d] protocol	N/A[e]
Marschollek et al, 2017 [31], Germany	Mobile device for chronic disease management	LMIC	To examine the mHealth literature to identify the technology most appropriate for LMIC	Literature review (n=255)	The amount of mHealth solutions in high-income country surpassed LMICs. SMS[f] text messages were highly used in LMICs compared with smartphone apps. SMS used for health promotion, monitoring, and management
Alghamdi et al, 2015 [29], N/A	mHealth technologies	Developing countries/LMIC	To describe opportunities and challenges in using mHealth in developing countries	Systematic review (n=27)	mHealth solutions were found to help with patient education, health promotion, disease management, and remote monitoring. mHealth technologies were limited in effectiveness because of the lack of interoperability, evaluation stands, and technology infrastructure present
Banbury et al, 2014 [34], Australia	Telehealth and eHealth[g] solutions	Rural residents of Australia and Aboriginals	To identify evidence on eHealth tools for disease management in remote areas	Rapid review (n=19)	Aboriginals feared the use of nontraditional tools referred to as *high-tech* treatments. Telehealth was increasingly used for remote consultation, but community consultation and collaboration was the key to solutions that were well adopted
Beratarrechea et al, 2017 [32], Argentina	mHealth intervention for NCDs[h]	LMIC	To identify the impact of mHealth tools on NCD outcomes in LMICs	Systematic review (n=108)	SMS was the most common and effective method to increase treatment adherence and health education. Effect of mHealth was positive but not as strong in LMIC for changing chronic disease outcomes
Bradford et al, 2015 [9], Australia	mHealth cardiac rehab program	Indigenous and Torres Strait Islanders	To customize cardiac program for Indigenous Australians	mHealth app co-design	App was customized according to rehab program, service delivery, and education formats. Changes were made to reflect cultural components by simplifying text, framing education in appropriate context, and adding Indigenous artwork

Brazionis et al, 2017 [38], Australia	Baseline retinal imaging (for telehealth screening); lifestyle survey; electronic CVD and diabetes decision support tool; mobile tablet (developed to enhance participant engagement in self-management)	Indigenous and Torres Strait Islanders	To investigate the efficacy of technology-facilitated CVD care in high-risk vulnerable Aboriginal and Torres Strait Islanders in remote/very remote Australia	Pre-post study design	N/A
Crengle et al, 2014 [33], New Zealand	3 educational sessions over 4 weeks with customized CVD medication program for Indigenous; tablet app for education session support and customized pill card for patient medication	Indigenous	To examine how customized program, delivered by health care professionals, will affect patient health literacy and CVD management	Design and protocol of RCT	Health literacy is a major barrier for medication adherence and CVD management. This barrier needs to be accommodated for, especially in Indigenous populations that have unique conditions and adult literacy challenges
Hamilton et al, 2018 [35], Australia	Smartphone for rehab and management of HF[i]	Indigenous	To evaluate how smartphone interventions for HF management could be utilized for cardiac patients in rural and remote settings	Systematic review (n=9)	mHealth delivery has potential to improve HF management of patients unable to attend traditional center programs. There are limited studies to evaluate its effectiveness in Indigenous communities. However, as most Indigenous people reside in remote areas, mHealth apps have greater potential to assist them
Parker et al, 2018 [36], Australia	mHealth and telehealth tools for CVD management	Vulnerable populations/ LMIC	To evaluate the benefit of using electronic, mobile, or telehealth tools for CVD management	Systematic review (n=18)	Reported intervention success varies according to health literacy level. Self-care was promoted with telephone or other mobile interventions. Social persuasion and goal setting were dominant factors influencing self-care. Vulnerable populations need tools that accommodate to their literacy level and that are supported by their social/cultural group

Yeates et al, 2017 [4], Canada	Text message intervention and smartphone BP monitoring	Indigenous and LMIC	To assess the effectiveness of SMS text message and community electronic BP measurement transfer to manage hypertension and in turn improve CVD-related outcomes	RCT protocol	This study is expected to provide insight into how innovative solutions can help assist Indigenous communities. Positive results will then be able to be transferable for application in other rural and low-resource settings

[a]CVD: cardiovascular disease.
[b]LMIC: low- and middle-income country.
[c]mHealth: mobile health.
[d]RCT: randomized controlled trial.
[e]N/A: not applicable.
[f]SMS: short message service.
[g]eHealth: electronic health.
[h]NCD: noncommunicable disease.
[i]HF: heart failure.
